# Physical embodiment and anthropomorphism of AI tutors and their role in student enjoyment and performance

**DOI:** 10.1038/s41539-024-00293-z

**Published:** 2025-01-08

**Authors:** Helene Ackermann, Anja Henke, Johann Chevalère, Hae Seon Yun, Verena V. Hafner, Niels Pinkwart, Rebecca Lazarides

**Affiliations:** 1 Department of Educational Sciences, University of Potsdam, Karl-Liebknecht-Straße 24/25, 14476 Potsdam, Germany; 2 Science of Intelligence, Research Cluster of Excellence, Marchstraße 23, 10587 Berlin, Germany; 3 Laboratoire de Psychologie Sociale et Cognitive (LAPSCO) UMR 6024, CNRS & Université Clermont-Auvergne, 17 Rue Paul Collomp, 63000 Clermont-Ferrand, France; 4 Humboldt-University Berlin, Unter den Linden 6, 10099 Berlin, Germany; 5 Deutsches Forschungszentrum für Künstliche Intelligenz (DFKI), Alt-Moabit 91c, 10559 Berlin, Germany

**Keywords:** Education, Psychology

## Abstract

Rising interest in artificial intelligence in education reinforces the demand for evidence-based implementation. This study investigates how tutor agents’ physical embodiment and anthropomorphism (student-reported sociability, animacy, agency, and disturbance) relate to affective (on-task enjoyment) and cognitive (task performance) learning within an intelligent tutoring system (ITS). Data from 56 students (*M* = 17.75 years, SD = 2.63 years; 30.4% female), working with an emotionally-adaptive version of the ITS “Betty’s Brain”, were analyzed. The ITS’ agents were either depicted as on-screen robots (condition A) or as both on-screen avatars and physical robots (condition B). Physical presence of the tutor agent was not significantly related to task performance or anthropomorphism, but to higher initial on-task enjoyment. Student-reported disturbance was negatively related to initial on-task enjoyment, and student-reported sociability was negatively related to task performance. While physical robots may increase initial on-task enjoyment, students’ perception of certain characteristics may hinder learning, providing implications for designing social robots for education.

## Introduction

As technology continues to advance and applications of new technologies and artificial intelligence (AI) in education are also developing further, they are reshaping learning and instruction^1^. This transformation provides both unprecedented opportunities and challenges for teachers, students, and schools^2,3^. However, given the rapid advances in AI development, there is an increasing need for comprehensive empirical research that thoroughly investigates the strengths and challenges of its application^1^. Particularly in education, evidence-based knowledge about these technologies and their consequences is crucial to ensure informed decision-making when discussing their application in everyday school practice^4^ and thus provide suitable learning opportunities for learners^5^. While some AI-based technologies for education, such as intelligent tutoring systems^6^ or conversational chatbots^7^, are well established and have been studied for several years, others have gained increasing attention only in recent years (e.g., generative AI^3^, social robots^8^, or holographic telepresence^9^). Consequently, research has provided evidence for the effective use of some AI-based technologies, while others have not yet been sufficiently explored. Additionally, novel approaches that involve combining multiple AI-based technologies to improve learning are under-researched and often suffer from methodological limitations, such as relying on small sample sizes, studying single cases, or predominantly including adults^1,10,11^. Therefore, these approaches may not adequately inform researchers about student learning in school contexts. The present study thus aims to address these methodological issues by empirically investigating how implementing and combining certain AI-based technologies in a real-world school setting relates to learning. It focuses on school-aged students and the integration of multiple AI-based technologies, contributing to the ongoing development of research on how these technologies can be effectively combined and applied in real-world educational settings to improve learning outcomes. In particular, the study addresses the use of social robots in combination with on-screen applications with school-aged populations in classroom environments.

One of the well-researched technologies utilizing AI in education is the intelligent tutoring system (ITS)^6^. An ITS is a computer-based learning environment providing AI-based, personalized learning opportunities by modeling and adapting to, for example, psychological and cognitive states of the learner^12^. As the use of ITSs allows multiple students to engage individually with a task and receive personalized learning content and feedback, it can be an effective means of classroom instruction^5^. Studies have shown that across various educational levels and a wide range of topics (e.g., physics, language learning, reading comprehension), ITSs can effectively foster student learning and improve students’ understanding of the subject^12,13^. Although they may not surpass individualized human tutoring, research indicates that ITSs can outperform other traditional modes of teaching, such as individual use of textbooks or teacher-led instruction in large groups^12^. Therefore, especially in large classes with a high diversity of learners, the use of ITSs can be an effective approach to providing suitable individual learning opportunities and thus account for individual student needs^5^.

Typically, ITSs adapt to learners’ cognitive learning, such as their aptitude and knowledge^6^. However, as Pekrun’s^14^ control-value theory suggests and research has evidenced^15,16^, learning does not only occur on a cognitive level. Rather, learning is specifically facilitated by the learning context, prior domain knowledge, and emotional experiences^14^. In accordance with control-value theory^14^, positive emotions in learning situations are associated with better learning outcomes and enhanced persistence, effort, and motivation to learn^15^, whereas negative emotions such as hopelessness, anxiety, or boredom can be associated with poorer learning outcomes, task-irrelevant thinking, and lower motivation to learn^16^. Emerging developments and research in AI-based learning and instruction acknowledge this relevance of students’ emotions while learning and increasingly incorporate affect-focused components with the aim of fostering a more positive emotional experience during learning^17–19^. Consequently, affective ITSs that can recognize and adjust to students’ emotional states are gaining more attention as well^20^. For example, one emotionally-adaptive ITS utilizing facial emotion recognition as well as text and speech analysis to adapt to learners’ emotional states demonstrated the potential to motivate learners and increase their satisfaction with the learning technology^21^. However, as automated measures of emotions, like facial recognition, lack predictive validity and have shown poor agreement rates with self-reports^22^, other studies rely on the experience sampling method (ESM), which collects longitudinal data by repeatedly surveying participants about their current emotions or motivational states^23^. In educational science, ESM is a reliable and validated approach for capturing students’ emotional states via self-report^23^. Using self-assessment methods to collect ground-truth data on students’ emotions has been highlighted as highly effective, thereby enhancing the accuracy of emotionally-adaptive tutoring systems^20^. For example, prior work used emotional information assessed through self-reported experience sampling to adapt the hint delivery strategy in the ITS “Betty’s Brain”^24^. Results showed that this emotionally-adaptive version improved the learning experience of higher achievers by reducing their boredom^25^. Thus, based on control-value theory^14^ and empirical findings, emotionally-adaptive ITSs can be seen as promising tools providing AI-based, well-tailored, and adaptive learning opportunities in educational settings. The present study focuses on one such emotionally-adaptive ITS and its consequences for learning outcomes.

Several modern educational technologies, including ITSs, incorporate virtual pedagogical agents as central design aspects. These animated agents can serve different roles and enable direct interaction with the learner^26^. Animated tutor agents, for example, have the advantage of including a social component into ITSs^27^ and keeping students from gaming the system^27^. When virtual pedagogical agents were introduced as a transformative learning paradigm around the turn of this century, they promised to significantly improve student engagement and motivation by creating rich, face-to-face learning interactions^28^. Nevertheless, researchers who synthesized the effect of virtual pedagogical agents on learner motivation and learning outcomes found no significant differences between agent and no-agent groups in the majority of the studies they reviewed in 2011^29^. On the contrary, another more recent meta-analysis focusing on studies published between 2012 and 2019 observed an overall small effect when comparing task performance in multimedia environments of students learning with a virtual agent versus those without an agent^30^. This, in contrast to earlier findings, suggests an advantage and increased instructional effectiveness of including pedagogical agents in digital learning environments like ITSs. As the meta-analysis covered more recent studies, we interpret this development as possibly reflecting technological advancements over the past decade, which may have contributed to the improved efficacy of modern virtual pedagogical agents.

A different approach that has emerged in the last decade is the physical embodiment of pedagogical agents, often realized through social robots^31^. At a conceptual level, the physical presence of artificial tutor agents allows for the inclusion of nonverbal cues and therefore greater immediacy of the agents, which is relevant for learners’ motivation and emotion^32,33^. In addition, physically present pedagogical agents may facilitate a more natural form of interaction, as they enable learners to interact with their physical environment and encourage social interaction^31^. Therefore, they may be seen as more effective compared to most other technological approaches in AI-based learning, like virtual agents or instructive virtual games^10^. Although a number of studies indeed suggest that physically embodied robots can enhance learning outcomes^31,34,35^ and are associated with increased enjoyment of learning^36^, other studies suggest that physical robots in education may not offer significant benefits in comparison to virtual on-screen agents^36,37^.

Nonetheless, research has shown that physical robots are perceived as more anthropomorphic, meaning human-like, than their virtual representations^38,39^, which may positively impact learning. Anthropomorphism can be defined as the attribution of human-like characteristics, motivations, intentions, or emotions to nonhuman agents and can influence the interaction between humans and technological agents^40^. Prior research has shown that there are interpersonal variations in how humans perceive and anthropomorphize robots that are designed to be human-like, and that these variations are influenced by factors such as age, personality, and thinking style^41^, which in turn shape trust and interaction dynamics^42,43^. For instance, younger students tend to anthropomorphize robots more and experience greater enjoyment in interactions^44^. Further, individual characteristics such as loneliness or anxiety may increase anthropomorphism^41^. Moreover, prior experience with robots relates to how learners perceive robots. More precisely, students with less experiences with robots are more likely to view robots as living, whereas those with more experiences better distinguish them from living entities^44^. Therefore, based on individual variations or prior expectations, anthropomorphism can impact learning in both positive and negative ways^45,46^. On the one hand, anthropomorphism of technology in education can improve its effectiveness^44,47^ and the perception of positive human-like characteristics of an artificial tutor might positively influence learning outcomes^48^. On the other hand, anthropomorphism might hinder learning through inducing fear, creating a rejection of the technology, or causing disappointment due to unmet expectations^49,50^. For instance, when robots simultaneously appear human-like and artificial, they can evoke fear or rejection^45^. These individual variations suggest that the degree of anthropomorphism and its effects on learning depends not only on a robot’s design but also on the characteristics and experiences of the learner interacting with the robot.

Research suggests that anthropomorphism can shift over time, depending on prior experience with robots^44^. Therefore, anthropomorphism might change with repeated interactions. As studies on social robots are often limited to a short duration, it is not yet clear how much of the results regarding perceptions and learning outcomes are related to novelty effects^51^. Although there is research on long-term interactions also discussing novelty^52^, implementing long-term interactions between humans and robots in research is often difficult and therefore few studies are available. Long-term interactions are particularly relevant for understanding anthropomorphism and the effectiveness of social interactions with robots. Although recent research suggest that anthropomorphism may be stable over shorter timescales compared to constructs like trust^53,54^, it is important to note that perceptions can still fluctuate rapidly in short interactions. Novelty may still play an important role in short-time interactions, as perceptions of robotic agents can change even within a few minutes in the same interaction^55,56^. Further, critical capabilities like empathy, which are relevant for strong connections and more natural interactions with robots, are affected from time^57^. Still, most studies evaluating empathic virtual agents or robots involve participants interacting with these agents for a short period or in a single session^57^. Since authors often do not disclose how their results may have been impacted by the novelty effect or to what extent they controlled for it, it is difficult to interpret whether changes in learning were influenced by the initial excitement caused by the new technology^10^. Thus, notable research gaps remain in the field of AI-based pedagogical agents and social robots as tutors in digital learning environments. In particular, knowledge about motivational-affective processes when learning with physically embodied pedagogical agents in addition to virtual ones remains limited, while accounting for possible novelty effects.

The present study contributes to an understanding of these issues and aims to advance the understanding of the impact of physically embodied social robots on learning outcomes. Specifically, we focused on differences in students’ on-task enjoyment and task performance when learning with two different adaptive systems: an AI-based ITS with virtual agents only or an AI-based ITS with physically embodied robots in addition to the virtual avatars. To investigate this, we conducted an experiment with a sample of *N* = 56 students using an emotionally-adaptive version of the ITS “Betty’s Brain”^24^. The students were divided into two conditions. In condition A, the robotic tutor agent was presented solely as a virtual on-screen avatar, while in condition B it was additionally physically present along with the virtual on-screen avatar (condition B). By comparing the two conditions, we aimed to answer the following research questions:

First, how does the physical presence of the robotic tutor agent influence students’ on-task enjoyment, task performance, and student-reported anthropomorphism of the agent (RQ1)? Second, how does anthropomorphism, more concretely student-reported sociability, animacy, agency, and disturbance of the robotic tutor agent, relate to both on-task enjoyment and task performance (RQ2)? We hypothesized that the physical presence of the robotic tutor agents in combination with the virtual avatar (condition B) would enhance students’ on-task enjoyment (H1a), anthropomorphism (H1b), and task performance (H1c) more than the virtual agents only (condition A). Additionally, we expected the positive anthropomorphic characteristics of student-reported sociability, animacy, and agency to be positively related – and the characteristic of disturbance to be negatively related – to both on-task enjoyment (H2a) and task performance (H2b).

## Results

### Descriptive statistics and correlations

Descriptive statistics are presented in Table 1 and bivariate correlations between all study variables, using Pearson correlation coefficients, are shown in Table 2. There was no significant correlation between the physical presence of the robotic tutor (condition A vs. condition B) and students’ on-task enjoyment, student-reported anthropomorphic characteristics of the robotic tutor agent, and task performance. However, on-task enjoyment during the first half (novelty phase) and second half (working phase) of the experimental session was significantly and negatively associated with the student-reported perception of the anthropomorphic characteristic of disturbance. Task performance and student-reported sociability of the robotic tutor agent were significantly and negatively correlated. Further, students’ on-task enjoyment in the novelty phase was strongly positively correlated with that in the working phase, but no significant correlation between on-task enjoyment and task performance was found. Additionally, better German grades were significantly associated with lower student-reported perceptions of disturbance by the robotic tutor agent. Gender significantly correlated with task performance, with male students performing better.

**Table 1:** Descriptive statistics

	Total		Condition A		Condition B		*d*_ΔConditon A vs_. _B_
	*M*	*SD*	*M*	*SD*	*M*	*SD*	
Age	17.75	2.63	17.66	2.66	17.85	2.64	−0.07
German grade _recoded_^a^	4.13	0.90	4.45	0.74	3.78	0.93	0.80
Student-reported sociability	4.36	1.27	4.34	1.30	4.39	1.26	−0.04
Student-reported animacy	3.01	1.31	2.95	1.40	3.07	1.22	−0.10
Student-reported agency	4.30	1.07	4.39	1.04	4.19	1.11	0.19
Student-reported disturbance	2.75	1.57	2.64	1.55	2.85	1.60	−0.13
Task performance^b^	0.51	0.19	0.47	0.17	0.54	0.20	−0.37
On-task enjoyment _novelty phase_	1.91	0.93	1.76	0.92	2.06	0.93	−0.33
On-task enjoyment _working phase_	1.65	1.01	1.59	1.01	1.72	1.03	−0.13

Descriptive statistics for all study variables for the total sample and the two conditions. In condition A, students learned only with the virtual avatars; in condition B the robotic tutor was also physically present.

*d*_ΔConditon A vs. B_ = Cohen’s d measuring the effect size of the difference between condition A and condition B. It is calculated as the difference in means (mean A - mean B) divided by the pooled standard deviation.

^a^The variable “German grade” was coded as follows: 1 = insufficient (worst grade) to 6 = very good (best grade).

^b^Task performance was measured as progress in “Betty’s Brain”.

**Table 2:** Bivariate correlations for analyzed variables

		1	2		3	4		5	6	7	8	9	10
1.	Condition^a^	—											
2.	German grade _recoded_^b^	−0.38**	—										
3.	Student-reported sociability	0.02	0.13		—								
4.	Student-reported animacy	0.05	0.10**			—							
5.	Student-reported agency	−0.09	0.22		0.59**	0.49**		—					
6.	Student-reported disturbance	0.07	−0.32*		−0.06	−0.10		0.04	—				
7.	Task performance^c^	0.18	−0.09		−0.29*	−0.15		−0.05	−0.11	−			
8.	On-task enjoyment _novelty phase_	0.17	0.13		0.23	0.25		0.26	−0.31*	0.26	—		
9.	On-task enjoyment _working phase_	0.07	0.16		0.27	0.20		0.27	−0.34*	0.13	0.63**	—	
10.	Gender^d^	−0.25	0.21		0.02	−0.08		−0.09	0.12	−0.30*	−0.14	−0.18	—

*p < 0.05, **p < 0.01.

^a^The variable “Condition” was coded as follows: 0 = condition A (no physical presence of the robotic tutor) and 1 = condition B (physical presence of the robotic tutor).

^b^The variable “German grade” was coded as follows: 1 = insufficient (worst grade) to 6 = very good (best grade).

^c^Task performance was measured as progress in “Betty’s Brain”.

^d^The variable “Gender” was coded as follows: 0 = male and 1 = female. Gender was included in the correlation analysis, but because it was not of theoretical interest for this study, it was not included in the statistical analyses.

### Physical presence of the robotic tutor agent

**Fig. 1:**
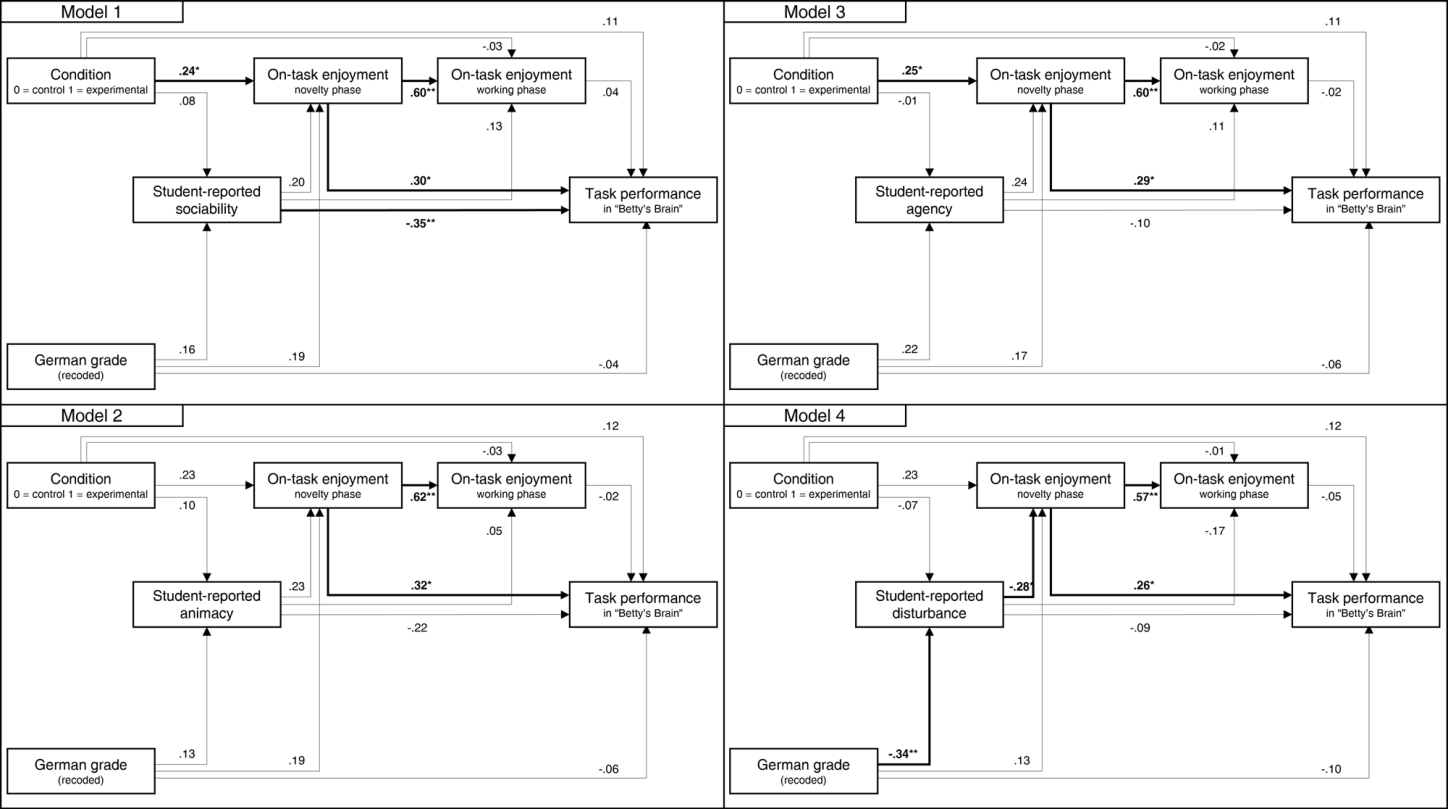
Results of the four path models. Each representing a subconstruct of the HRIES scale. Bold lines indicate statistically significant (*p* < 0.05) standardized coefficients (*p < 0.05, **p < 0.01). Thin lines indicate included paths that are non-significant.

To test our hypotheses of group-related differences in on-task enjoyment (H1a), anthropomorphism (H1b), and task performance (H1c), we specified four different path models, one for each subdimension of anthropomorphism of the Human-Robot Interaction Evaluation Scale (HRIES)^46^. The coefficients of all four models are reported in Table 3 and visualized in Fig. 1. All path models demonstrated good model fit (Model 1_Soc_: *χ²* = 0.19, df = 1, *p* = 0.666, CFI = 1.00, TLI = 1.00, RMSEA < 0.001, SRMR = 0.01; Model 2_Anim_: *χ²* = 0.27, df = 1, *p* = 0.606, CFI = 1.00, TLI = 1.00, RMSEA < 0.001, SRMR = 0.01; Model 3_Agen_: *χ²* = 0.21, df = 1, *p* = 0.650, CFI = 1.00, TLI = 1.00, RMSEA < 0.001, SRMR = 0.01; Model 4_Dist_: *χ²* = 0.05, df = 1, *p* = 0.822, CFI = 1.00, TLI = 1.00, RMSEA < 0.001, SRMR = 0.01). Although the correlations did not indicate a significant association between the physical presence of the robotic tutor and on-task enjoyment, in two out of four path models the coefficient between the condition and on-task enjoyment in the novelty phase was significant, while the other two models showed not significant associations with *p* = 0.061 and *p* = 0.054 (H1a). In condition B, students reported higher on-task enjoyment than in condition A. However, learning with or without a physically present robot along with a virtual agent (condition A vs. condition B) and on-task enjoyment during the working phase were not significantly related across all models (H1a). Furthermore, the path models did not reveal any significant associations between the robotic tutor agents’ physical presence and the student-reported perceptions of its sociability, animacy, agency, or disturbance (H1b). Also, all path models consistently showed no significant relation between the tutor’s physical presence and students’ task performance (H1c).

**Table 3:** Results of the path models

	*ß*	*SE*	*95%CI*	*p*
Model 1: Including HRIES subscale ‘sociability’				
Student-reported sociability				
Condition	0.08	0.14	[−0.20, 0.36]	0.590
German grade _recoded_	0.16	0.13	[−0.10, 0.42]	0.236
On-task enjoyment _novelty phase_				
Student-reported sociability	0.20	0.12	[−0.03, 0.42]	0.087
Condition	0.24	0.12	[−0.01, 0.46]	0.042
German grade _recoded_	0.19	0.14	[−0.08, 0.46]	0.171
On-task enjoyment _working phase_				
Student-reported sociability	0.13	0.10	[−0.06, 0.33]	0.185
Condition	−0.03	0.10	[−0.23, 0.17]	0.776
On-task enjoyment _novelty phase_	0.60	0.11	[0.39, 0.80]	0.000
Task performance				
Student-reported sociability	−0.35	0.09	[−0.53, −0.17]	0.000
Condition	0.11	0.14	[−0.17, 0.40]	0.426
On-task enjoyment _novelty phase_	0.30	0.12	[0.07, 0.53]	0.010
On-task enjoyment _working phase_	0.04	0.13	[−0.22, 0.30]	0.766
German grade _recoded_	−0.04	0.13	[−0.30, 0.22]	0.753
Model 2: Including HRIES subscale ‘animacy’				
Student-reported animacy				
Condition	0.10	0.15	[−0.20, 0.40]	0.518
German grade _recoded_	0.13	0.13	[−0.13, 0.39]	0.313
On-task enjoyment _novelty phase_				
Student-reported animacy	0.23	0.13	[−0.03, 0.48]	0.080
Condition	0.23	0.12	[−0.01, 0.47]	0.061
German grade _recoded_	0.19	0.15	[−0.09, 0.48]	0.182
On-task enjoyment _working phase_				
Student-reported animacy	0.05	0.10	[−0.14, 0.24]	0.613
Condition	−0.03	0.11	[−0.24, 0.17]	0.757
On-task enjoyment _novelty phase_	0.62	0.11	[0.41, 0.82]	0.000
Task performance				
Student-reported animacy	−0.22	0.11	[−0.45, 0.00]	0.050
Condition	0.12	0.14	[−0.16, 0.39]	0.415
On-task enjoyment _novelty phase_	0.32	0.13	[0.07, 0.57]	0.013
On-task enjoyment _working phase_	−0.02	0.14	[−0.29, 0.26]	0.895
German grade _recoded_	−0.06	0.13	[−0.31, 0.19]	0.626
Model 3: Including HRIES subscale ‘agency’				
Student-reported agency				
Condition	−0.01	0.14	[−0.28, 0.25]	0.938
German grade _recoded_	0.22	0.13	[−0.03, 0.46]	0.079
On-task enjoyment _novelty phase_				
Student-reported agency	0.24	0.12	[0.00, 0.48]	0.050
Condition	0.25	0.12	[0.02, 0.48]	0.033
German grade _recoded_	0.17	0.15	[−0.12, 0.45]	0.256
On-task enjoyment _working phase_				
Student-reported agency	0.11	0.12	[−0.13, 0.34]	0.375
Condition	−0.02	0.11	[−0.23, 0.19]	0.852
On-task enjoyment _novelty phase_	0.60	0.12	[0.37, 0.82]	0.000
Task performance				
Student-reported agency	−0.10	0.15	[−0.39, 0.19]	0.506
Condition	0.11	0.15	[−0.19, 0.40]	0.475
On-task enjoyment _novelty phase_	0.29	0.13	[0.03, 0.54]	0.029
On-task enjoyment _working phase_	−0.02	0.15	[−0.31, 0.28]	0.908
German grade _recoded_	−0.06	0.14	[−0.32, 0.20]	0.659
Model 4: Including HRIES subscale ‘disturbance’				
Student-reported disturbance				
Condition	−0.07	0.13	[−0.31, 0.19]	0.612
German grade _recoded_	−0.34	0.11	[−0.55, −0.13]	0.001
On-task enjoyment _novelty phase_				
Student-reported disturbance	−0.28	0.12	[−0.52, −0.03]	0.026
Condition	0.23	0.12	[0.00, 0.47]	0.054
German grade _recoded_	0.13	0.14	[−0.15, 0.40]	0.361
On-task enjoyment _working phase_				
Student-reported disturbance	−0.17	0.09	[−0.34, 0.01]	0.071
Condition	−0.01	0.10	[−0.21, 0.19]	0.903
On-task enjoyment _novelty phase_	0.57	0.11	[0.35, 0.79]	0.000
Task performance				
Student-reported disturbance	−0.09	0.13	[−0.34, 0.15]	0.452
Condition	0.12	0.15	[−0.17, 0.40]	0.425
On-task enjoyment _novelty phase_	0.26	0.13	[0.00, 0.51]	0.046
On-task enjoyment _working phase_	−0.05	0.15	[−0.34, 0.23]	0.717
German grade _recoded_	−0.10	0.14	[−0.37, 0.17]	0.474

### Anthropomorphism of the robotic tutor agent

Using the same four models described above, we investigated the relationships between anthropomorphism and both on-task enjoyment (H2a) and task performance (H2b). Out of the four subdimensions of anthropomorphism, only student-reported disturbance (see Model 4_Dist_ in Table 3 and Fig. 1) was significantly and negatively related to students’ on-task enjoyment during the novelty phase, such that a higher perception of disturbance was related to lower on-task enjoyment (H2a). None of the student-reported anthropomorphic characteristics revealed a significant association with on-task enjoyment in the working phase (H2a). Further, student-reported animacy, agency, and disturbance of the robotic tutor did not relate to students’ task performance (H2b). However, as shown in Model 1_Soc_ (Table 3 and Fig. 1), student-reported sociability was significantly and negatively associated with task performance (H2b).

### Secondary findings

Across all four models, on-task enjoyment in the novelty phase was significantly and positively associated with both on-task enjoyment in the working phase and task performance. However, on-task enjoyment during the working phase was not significantly related to task performance. Students’ German grades were associated with neither on-task enjoyment in the novelty phase nor their task performance. Nevertheless, the student-reported perception of disturbance was significantly and negatively associated with the German grade as the only one of the four anthropomorphic characteristics (see Model 4_Dist_ in Table 3 and Fig. 1). Thus, students with better German grades perceived the robotic tutor agent as less disturbing.

## Discussion

This study investigated the role of the physical embodiment of a pedagogical tutor agent of an AI-based adaptive system in addition to its virtual avatar in emotional and cognitive learning outcomes and students’ perceptions of the tutor agent’s anthropomorphic characteristics. In addition, the relevance of anthropomorphic characteristics for students’ emotional and cognitive learning was investigated. Key findings indicate that the physical (non-)presence of the pedagogical agent was not significantly associated with either (1) student-reported anthropomorphism or (2) task performance. However, (3) when physically present, the robotic tutors were significantly related to higher on-task enjoyment in the first half of the interaction (novelty phase) in two of the four models (Model 1_Soc_ and Model 2_Anim_). Further, (4) a higher student-reported perception of sociability was associated with lower task performance, and (5) a higher student-reported perception of disturbance was related to lower on-task enjoyment during the novelty phase. In the following sections, we discuss these findings along with our hypotheses and specify potential implications for the implementation of social robots as AI-based tutors in learning and education, while acknowledging the limitations of our study, including the small sample size and specific experimental setup.

Contrary to our initial hypothesis, the physical presence of the robotic tutor agent did not relate to either higher anthropomorphism of the robotic agent (H1b) or task performance (H1c). Thus, the implementation of physical robots alongside virtual avatars did not lead to the tutors being perceived as more human-like, nor did it improve students’ progress on the task better than virtual avatars only. Although we partly observed higher initial on-task enjoyment among students working with the physical robot (H1a), this association occurred only in the first half of the experiment (novelty phase) and was not evident in the second half (working phase). This observation can be interpreted as a possible effect of initial novelty^51^. As social robots are still rather uncommon in everyday life, participants likely were interacting with a physical robot for the first time. The results indicate that although the physical presence of the robotic tutors seems to have evoked an initial excitement, this novelty factor vanished in the working phase. This could be attributed to the robot becoming a familiar presence over time^52^, allowing students to redirect their focus more to the actual task^10^.

It is important to note that, alternatively, the significant positive relation between the physical embodiment and on-task enjoyment in the novelty phase could potentially also be explained by factors related to the experimental setting. For example, students interacting with the physically embodied robots might have experienced more technological errors, which could have potentially increased negative and decreased positive emotions^58^, possibly limiting the impact of physical embodiment beyond the novelty phase. Additionally, the different modalities of interaction between the conditions could have played a role in students’ emotional experiences. In condition A, the virtual on-screen robotic tutor agent only provided text-based hints. In condition B, the physically embodied robot also incorporated gestures, and verbal communication, potentially leading to more distractions or a different engagement level that may have impacted students’ on-task enjoyment in the working phase compared to condition A. Moreover, the emotionally-adaptive version of the ITS used in both conditions of this study might have also influenced students’ emotional experiences during the learning session in general. The tutor agent provided hints based on students’ self-reported emotional states, which became more detailed when emotions indicated task disengagement and less detailed when students felt engaged or enjoyed the task. This responsive approach might have led to students with high initial enjoyment receiving only vague hints, potentially contributing to a decrease in their engagement and enjoyment over time.

However, these results need to be interpreted with caution, as the relation between the physical presence of the robotic tutor and the on-task enjoyment in the novelty phase was only significant in two of the four models and not present in the bivariate correlations. This discrepancy may be explained by possible third variables, as the path models take into account the effects of such factors as, for example, the HRIES subdimensions, while the bivariate correlations are not taking into account the possible influence of third variables. Thus, when the effect of anthropomorphism (HRIES) on on-task enjoyment is simultaneously considered in the model, a significant effect of the physical presence of the robotic tutor on on-task enjoyment in the novelty phase can be shown. Nevertheless, the role of the robotic tutor’s physical presence in motivational-affective learning outcomes remains inconclusive and requires further research.

Thus, the strengths of physical robots in increasing student learning outcomes and enjoyment found in prior research could not be replicated in the present study. Additionally, no effects on the perception of anthropomorphistic characteristics were observed. This finding contradicts previous research, which showed that text-based communication can lead to dehumanization, while speech with naturalistic cues can foster anthropomorphism^59^ as different communication styles or the quality of an agent’s voice can influence anthropomorphic perceptions, trust and performance^43,60,61^. However, this was not true for our study. Rather, the results of this study do not show clear positive outcomes for students’ learning from implementing physically embodied robotic tutors. While we did indeed partly observe a positive impact of physical robots on students’ on-task enjoyment, the presence of a novelty effect in the short 1 h experimental interaction time suggests that the positive effect on enjoyment may not persist in long-term settings. However, this study identified potential benefits of physical robots in terms of students’ initial enjoyment by acknowledging and accounting for possible novelty effects. While future research could focus on normalizing for novelty effects in the experimental setup, for example through warm-up periods where participants become familiar with the robots before the actual study begins, it might also focus on developing strategies for utilizing the novelty effect as a source of information when designing robots for educational purposes instead of treating it as a source of noise^62,63^.

Another key finding of this study, offering potential implications for the development of robotic tutor agents for educational purposes, relates to the consequences of anthropomorphism for students’ emotional and cognitive learning. In line with our hypothesis, we observed associations between the perceptions of anthropomorphic characteristics and students’ on-task enjoyment (H2a) and task performance (H2b). In each case, one specific subdimension indicated significant relevance. While higher student-reported disturbance was associated with lower on-task enjoyment in the novelty phase, higher perceived sociability was linked with lower task performance. Surprisingly and contrary to our initial assumptions, students’ progress on the task was lower when the robotic tutor agent was perceived as more sociable, irrespective of whether the robot was physically or virtually present. This contradicts previous research, which has proposed that human-like characteristics in robotic tutor agents have a beneficial effect on student learning and should be facilitated in technology^40^. Instead, the findings of this study suggest that if a robotic tutor is perceived as overly sociable, it may distract students from the task at hand rather than assist them in achieving better results. This coincides with the results of earlier studies, which found a social robot to have distracting and negative effects on participants’ performance^37,64^. This finding highlights the importance of considering that the way students perceive social robots can influence the degree to which they benefit from them. Additionally, our findings illustrate that certain human-like characteristics, such as perceived disturbance, can lead to decreased on-task enjoyment. Although perceived disturbance is the only dimension incorporating negative attributions, it remains a relevant subdimension of anthropomorphism, considering human-like traits related to the perception of danger and strangeness^46^. This finding aligns with the theoretical perspective that human-like features, if perceived negatively, can induce negative feelings and therefore detract students from learning. Overall, our results suggest that high anthropomorphism – negative as well as positive human-like traits – might not always be beneficial for learning and should be evaluated with respect to different subdimensions. Given the substantial interpersonal differences in how humans anthropomorphize, whether influenced by characteristics like age and personality^41^ or shaped by factors such as prior experience and unmet expectations^44^, it is crucial to balance designing robots with human-like traits and mitigating the potential negative effects of these perceptions. Our findings underscore the importance of more nuanced research when examining the relevance of anthropomorphism of robots within educational environments to optimize their effectiveness and support learning, while minimizing potential negative effects resulting from strong perceptions of human-likeness.

Consequently, the present study highlights that possibly not all students benefit equally from a robotic tutor, as their performance and enjoyment could be influenced by whether they feel sympathy towards the robot or find it intimidating. In addition to individual perception, the relevance of students’ prerequisites has been indicated too. In our study, we controlled for students’ self-reported, most recent term grade in German, which could be seen as a proxy for their German language proficiency. We observed that better German grades were significantly associated with lower student-reported disturbance, suggesting that students with lower German language proficiency may have perceived the robotic tutor agents as more disturbing. Whereas prior research has proposed that social robots can be particularly distracting for students with lower educational competencies^64^, the present study further suggests potential differences in the perception of such pedagogical agents. While working with the AI-based ITS “Betty’s Brain”^24^, students with lower German language proficiency may have already encountered difficulties with the learning task itself, which includes extensive reading and therefore reading comprehension. The interfering text-based hints of the robotic tutor, whether in written form from the virtual robot or in acoustic form from the physical robot, might further distract these students, increasing their difficulties while learning, and thus be counterproductive for the overall learning process. In these cases, students might benefit more from either a human tutor or a robotic tutor capable of adapting not only the detail but also the number and timing of hints to the students’ individual needs. Once more, these aspects are of relevance when evaluating the implementation of robotic tutors in learning and instruction as well as future research directions. However, it is important to note that these findings are based on a specific sample and experimental setup, and further research with diverse participant groups is needed to validate these results. Given that factors like age, personality, thinking style, and prior experience with robots are known to influence anthropomorphism^41,44^, future research should explore these individual differences in more detail, as these factors could provide valuable insights into whether our findings hold true for different participant groups.

It is important to acknowledge several limitations of the present study. Firstly, the robotic tutor agents used in this study had a limited, purely supportive role, providing hints according to a predefined matrix^25^. Although the hints’ level of detail was adapted to the students’ performance and emotional experience based on an AI algorithm, they were scheduled to be given every 10 min. During these time intervals, the robotic tutor agents remained mainly passive and only interacted in subtle ways, mostly (if at all) through the ITS’s interface by choice of the student. This design could have impacted the students’ perceptions of the robotic tutor agents’ anthropomorphic characteristics, as the level of the robots’ interactive behavior can influence anthropomorphism^65^. Secondly, it is worth noting that the human-robot interactions in the present study were of relatively short duration. The total interaction time of only 1 h is insufficient to assess long-term and retention effects. Additionally, the short interaction time raises questions about whether the positive impact of initial enjoyment in the novelty phase on the enjoyment in the working phase, which aligns with control-value theory^14^, can carry over for longer periods of time or whether this association subsides shortly after the initial interaction with the ITS and the robotic agent. Thirdly, while our study focused primarily on the robotic tutor agent, the robotic peer agent (Cozmo) did not play a significant interactive role in both conditions. The Cozmo robot was a peer-agent taught by each learner within the ITS. However, it did not engage in direct interaction with the students. This might have limited the understanding of the potential impact of peer-like social interactions on learning outcomes. As prior findings suggest, social interaction with tutee agents and treating them as partners can significantly enhance learning outcomes^66^. Future research could benefit from exploring the interactive roles of both tutor and peer agent in a more balanced manner, examining how direct interaction with both types of agents influences students’ learning outcome and experiences. Lastly, the sample size of only 56 students may, despite being a comparatively large sample size in the context of human-robot interaction studies, be considered relatively small for drawing overarching conclusions. Therefore, further research conducted over an extended period of time, including longer interaction periods and involving a more extensive sample, is necessary to gain a more comprehensive understanding of the implications of the physical presence of robot tutors in the educational context.

In conclusion, the present study contributes valuable findings to the current ambivalent state of research on physically embodied robotic tutors. In this study, additionally employing physical robots was not beneficial over on-screen avatars alone. The results also point to certain aspects of implementing robotic tutors in general that should be approached with caution and investigated further in more representative studies. More specifically, the results suggest that – irrespective of physical embodiment – when students perceive pedagogical agents as overly sociable, it may distract them from the task, resulting in a lower task performance. Perceiving the robotic agents as disturbing may reduce students’ on-task enjoyment. While these findings indicate potential drawbacks of anthropomorphism for learning, it is also possible that students who were already struggling with the task perceived the agents as more sociable or disturbing, rather than these perceptions directly causing the lower performance or enjoyment. Nevertheless, the initial improvement of on-task enjoyment observed with physical robotic tutors suggests that physical embodiment of AI-based pedagogical agents, in general, holds promise for students’ emotional experience while learning. In our view, a key challenge lies in finding ways to maximize this positive novelty effect to maintain the enjoyment of learning with a physical robotic tutor over the long term. Future research should try to generate effective long-term learning scenarios that go beyond the novelty effects, which this study could only account for to a limited extend. Moreover, it is important to consider students’ individual prerequisites when designing AI-based robotic tutor agents for educational practice. Given the interpersonal differences in how humans anthropomorphize, balancing the design of robots with human-like traits and mitigating potential negative effects is essential. Future research should explore these dimensions further, focusing on how different subdimensions of anthropomorphism influence learning outcomes and emotional experiences to avoid unintended drawbacks.

## Methods

### Sample description

Originally, a total of 64 students from two secondary schools in Germany participated in the experiment. Due to incomplete data, *n* = 6 participants had to be excluded from analysis as they did not provide any data at the pre-test measurement, and *n* = 2 had to be excluded due to extremely outlying age values (≥25 years), resulting in a sample of *N* = 56 students (*M*_age_ = 17.75, *SD*_age_ = 2.63; 30.4% female, 69.6% male). Participants were randomly assigned to one of the two experimental conditions. In condition A, supported only by a virtual robotic tutor agent, 51.8% of participants were included (*n*_con_ = 29 students: 41.4% female, 58.6% male). Meanwhile, condition B, in which students were additionally supported by a physically present robotic tutor agent, comprised 48.2% of participants (*n*_exp_ = 27 students: 18.5% female, 81.5% male). The majority of the participants were native German speakers (78.6%). Prior to participation, informed consent was obtained from all participants and, if required, their parents or legal guardians. The study was performed in line with the principles of the Declaration of Helsinki. It received approval from the University of Potsdam Ethics Committee (number 78/2021) as well as the Ministry of Education, Youth and Sports of the Federal State of Brandenburg (number 22/2022).

### Procedure

**Fig. 2:**
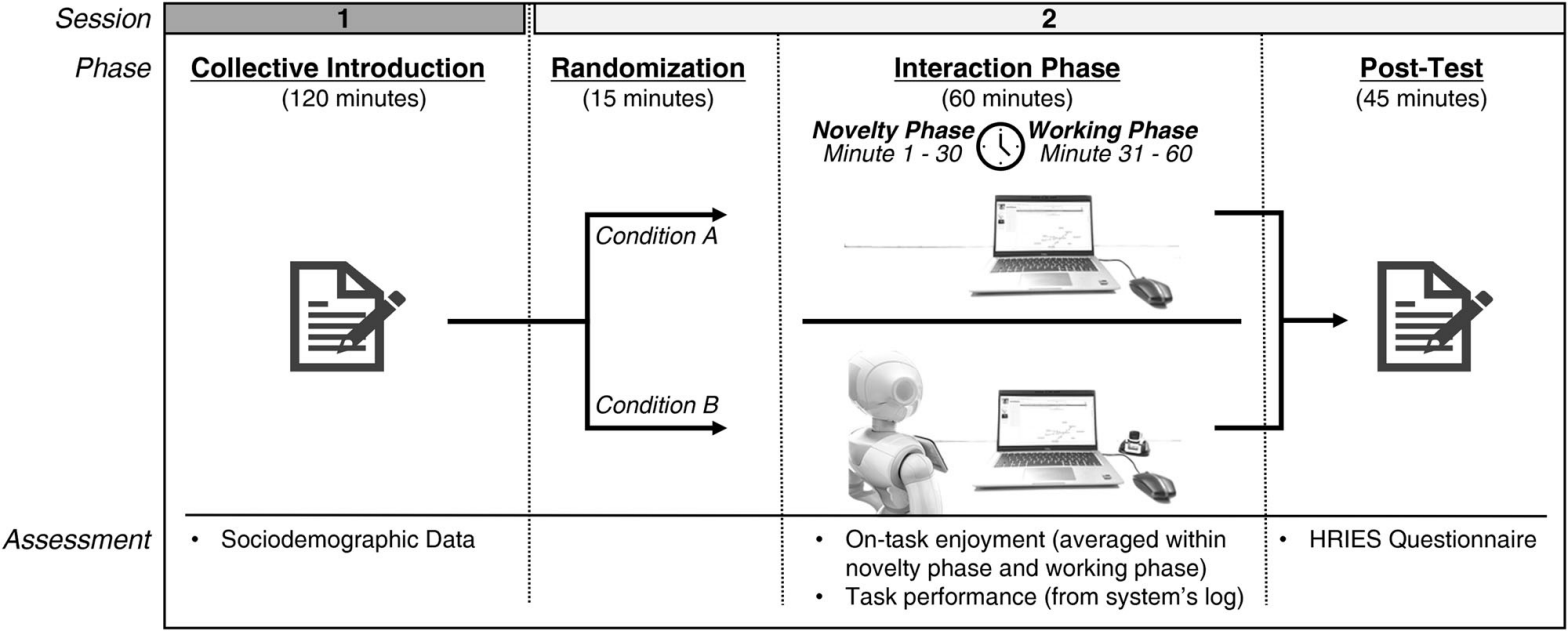
Visualization of procedure and experimental setup. The study consisted of two 2 h sessions. Participants in condition (**A**) were learning with an ITS with virtual robotic agents. In the condition (**B**), participants were additionally accompanied by physically present robotic agents. The experimental setup included a robotic peer agent (Cozmo robot) and a robotic tutor agent (Pepper robot).

As part of the participating schools’ science weeks, students participated in the study over two 2 h sessions. During the first session, sociodemographic data were assessed. Additionally, the students were verbally introduced to the computer-based learning environment, namely the ITS “Betty’s Brain”^24^, an open-ended learning platform designed to enhance students’ engagement in science phenomena^24^. During the second session, students spent 60 min on the individual learning task within the ITS, while the remaining time was used for the post-test, organizational aspects and answering participants’ questions (see Fig. 2 for a visualization of the procedure).

“Betty’s Brain” is a well-established ITS which follows a learning-by-teaching paradigm^67^. Students learn about specific scientific phenomena by reading a virtual science book and building a concept map to teach a virtual peer agent, usually called Betty, with the support from a virtual tutor agent, typically called Mr. Davis. In our case, students learned about climate change by teaching a virtual representation of a Cozmo robot instead of Betty, while being supported by a representation of a Pepper robot instead of Mr. Davis. The ITS usually follows a progressive hint delivery approach, with the tutor agent gradually providing students with more detailed hints as they experience consecutive failures. However, we used an emotionally-adaptive version of the ITS instead, which had been developed and translated to German in prior work^25,68^. In this version, students received hints based on their current self-reported emotional state. Every 10 min students selected the label that best represented their emotional state (“engaged concentration”, “enjoyment”, “confusion”, “frustration”, or “boredom”). These self-reports, together with the student’s performance, determined the hint provided from four levels of support, ranging from vague (e.g., “*I still think you need to spend more time reading the page called ‘Droughts and the Water Cycle’. There’s a causal relationship on that page that you haven’t taught Cozmo yet*”) to detailed information (e.g., “*You need to add the link “droughts” to the map. This portion of the science book page called water cycle explains the relationship. See if you can figure out which part of this passage explains the relationship between precipitation and vegetation*”). Specifically, hints were increasingly more detailed when students’ emotions were closer to task disengagement and the accuracy of their concept map for a chapter was ≤ 50%^25^. At each 10-min interval, one hint was given subsequent to the emotional assessment.

Thus, while working with the ITS, the students were prompted every 10 min to report their emotional states and subsequently received feedback from the robotic tutor agent (Pepper robot) to guide their learning process. The robotic tutor agent provided a hint every 10 min in both conditions but remained mostly passive in between these intervals. Students had the option to seek help from the tutor agent at any time by clicking an “Ask Pepper” button, which triggered responses to general questions like “*How do I build a concept map?*”. However, the tutor agent did not respond spontaneously to the learner outside of these pre-included questions, resulting in a primarily one-way interaction. The robotic peer agent (Cozmo robot) did not directly interact with the learner. Although it occasionally provided social comments, such as “*Thanks for teaching me about climate change!*” or “*Great! I got most of these questions right. We’ll be done learning soon!*” as text on screen within the ITS interface, it primarily acted as the character being taught by the learner. Cozmo was quizzed from time to time, reinforcing its role as a peer rather than that of a tutor.

As this study aims to investigate the influence of the physical embodiment of an ITS’ virtual pedagogical agents on learning outcomes, two conditions were compared. In condition A, both Pepper and Cozmo were only present as virtual on-screen avatars, while in condition B they were also physically present in the classroom (see Fig. 2 for an illustration). While the physically embodied robots incorporated movements and gestures while delivering hints (Pepper), moved while starting the task (Pepper and Cozmo), and demonstrated facial expressions (Cozmo), the virtual avatars were static representations of the respective robots. Another important difference between the conditions was the modality of speech: In condition B, the physical robotic tutor agent (Pepper) provided verbal hints by speaking them aloud. Conversely, in condition A, the hints were only presented as text on the computer screen. The robotic peer agent (Cozmo) communicated solely through text on the computer screen in both conditions.

After working with the ITS, students completed a post-test questionnaire on the computer screen, where they self-reported how they perceived the robotic agents’ anthropomorphic characteristics. Each agent was evaluated separately and sequentially, with students viewing pictures of the robotic agents presented in a randomized order.

### Measures

#### Anthropomorphism of robotic agents

Students were asked to evaluate the extent to which they attributed human-like qualities to the robots in the post-test questionnaire following their interaction with the ITS and the robotic agents. Students’ perceptions were assessed using the Human-Robot Interaction Evaluation Scale (HRIES), a questionnaire assessing anthropomorphism using a multicomponent approach^46^. Specifically, the HRIES incorporates multiple psychological dimensions based on dehumanization theory and psychosocial theory, offering a more comprehensive assessment. It measures the anthropomorphism of a robot using four dimensions: perceived sociability, animacy, agency, and disturbance. It has repeatedly shown good psychometric reliability and structural validity^46^. Each dimension is assessed on a subscale including four items with each item being significantly associated with its respective factor (*p* < 0.001)^46^. The sociability scale encompasses the items warmth, trustworthy, likable, and friendly. Animacy involves the items humanlike, alive, natural, and real. Agency includes intelligent, rational, intentional, and conscious. Lastly, disturbance includes the items scary, creepy, uncanny, and weird^46^.

#### On-task enjoyment

Students emotional experience was assessed through experience sampling (self-report) using an adapted, short version of the epistemically-related emotion scale^69^. Students were prompted about their emotional states every 10 min in the ITS. Besides selecting the label that best represented their state to fulfill the modalities of the emotionally-adaptive ITS, students also rated how strongly they were experiencing certain emotions (“How strongly are you experiencing enjoyment right now? Please click on the button that best describes the intensity”) on a 5-point Likert-type scale with possible responses ranging from “not at all” to “very strongly”. Although a selection of emotions represented in the epistemically-related emotion scale was assessed using single items, the present study only examines on-task enjoyment because it is the only positive activating achievement emotion with activity focus according to control-value theory^66^. Therefore, higher on-task enjoyment also reflects perceiving the task at hand as more controllable and valuable and is therefore associated with higher learning outcomes and performance. For the analysis, the students’ reported on-task enjoyment was split into a novelty phase and a working phase (30 min each) to account for a possible novelty effect^51^. This division was based on an a priori analysis, revealing significant changes in mean on-task enjoyment scores after 30 min of interaction across both conditions (t(52) = 2.18, *p* = 0.034). By distinguishing the two phases in our data analysis, we aimed to account for the changes in emotional experiences due to the novelty of the setup. The reported on-task enjoyment was averaged within each phase for the analysis.

#### Task performance

Students’ task performance was assessed by means of progress in the ITS “Betty’s Brain”^24^ and derived from the system’s log. In “Betty’s Brain”, learners are asked to develop a concept map representing their understanding of the topic, and their performance is evaluated based on how accurately their concept maps align with a fixed expert model^24^. The expert model used in this study has been developed by the original research group behind “Betty’s Brain” and is described in detail on page 202 of Kinnebrew and colleagues^70^. Task performance represents the percentage of progress the learner has made on this learning task, with 100% indicating the completion of the task, which entails creating a fully correct concept map. This includes correctly identifying causal relationships and representing them through accurate links between constructs. The system continuously assesses task performance and provides hints based on this measure during the session. For our analysis, we measured how closely students’ final concept maps, created at the end of the session, matched the expert model.

#### Additional measures

Since the ITS “Betty’s Brain”^24^ mainly relies on text and extensive reading, students’ self-reported, most recent German grade, which can be seen as a proxy for their German language proficiency, was assessed and included in the analysis as a control variable. For this purpose, students were asked to self-report the German grade they had received on their last school report (term level). Additionally, during the first session of the experiment, sociodemographic data, including age, gender, and the first language learned at home, were collected through self-report. These sociodemographic variables were not included, as they were not relevant to answering the research question of the present study.

### Statistical analysis

**Fig. 3:**
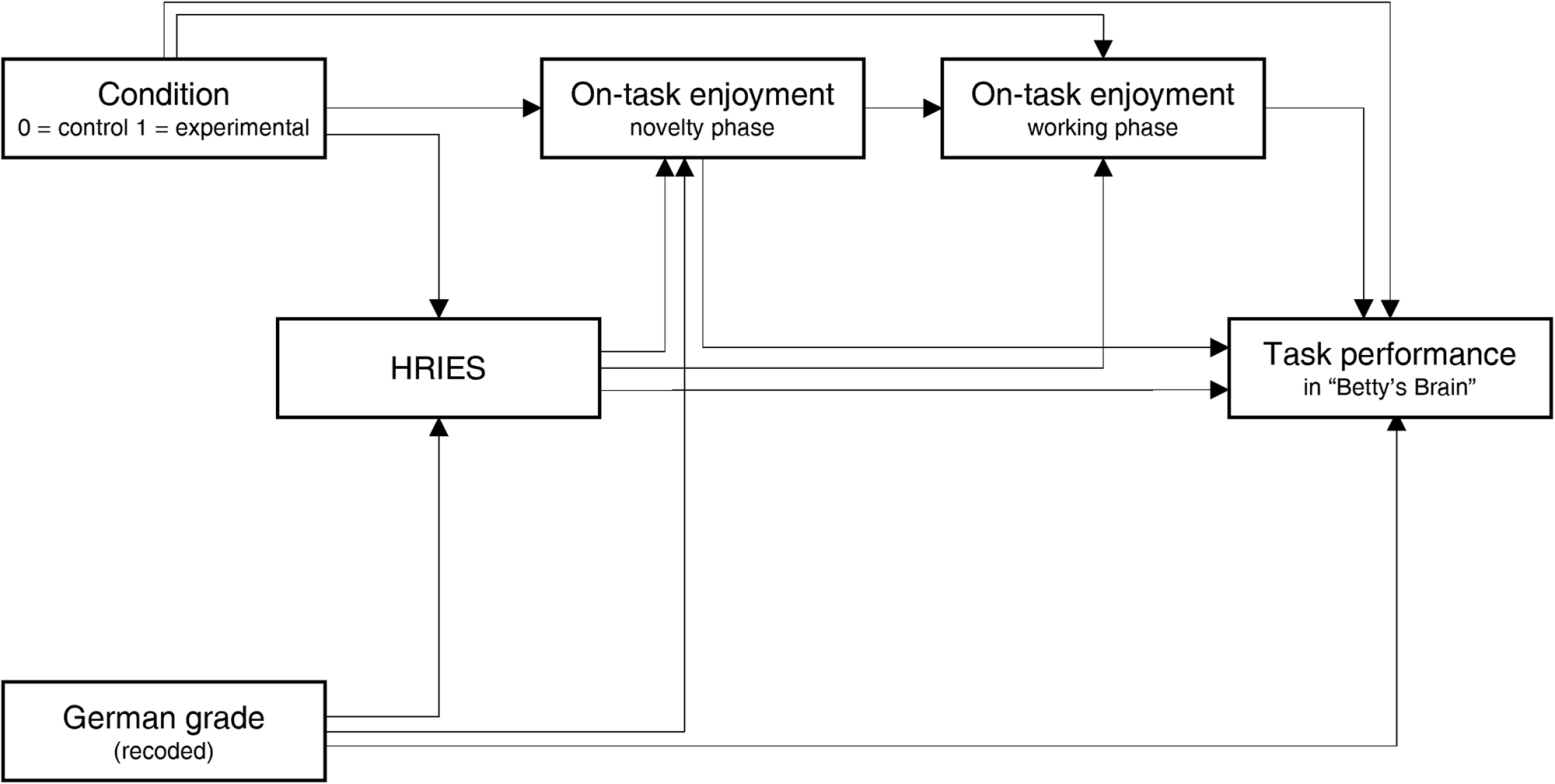
General path model structure of all four models. HRIES = human-Robot Interaction Evaluation Scale. One model was conducted for each of the four subscales of the HRIES (sociability, animacy, agency, and disturbance).

To test our hypotheses, we specified four separate path models. To reduce their complexity, we specified one model for each subdimension of the HRIES. As this study concentrates on physically embodied virtual tutors, only responses related to the robotic tutor agent (Pepper robot) were considered. The general structure of all four path models was the same and is illustrated in Fig. 3. In the models, the physical (non-)presence of the robotic tutor agent (condition) was regressed on the respective HRIES construct (sociability, animacy, agency, or disturbance), task performance, and on-task enjoyment in both the novelty and working phases (H1). Furthermore, each HRIES construct was regressed on on-task enjoyment in both the novelty and working phases, as well as task performance (H2). We controlled for the students’ German language proficiency in the form of the final German grade received for the last term, which was regressed on the respective HRIES construct, on-task enjoyment in the novelty phase, and task performance. For easier interpretation, the German grade was recoded so that higher values indicated higher grades. We decided against including gender in our path models, as it was not of theoretical interest and not expected to be relevant to answer our research questions. However, we subsequently found that there was a significant correlation between students’ gender and their task performance. To investigate this unexpected relation further, we included gender as a covariate in a previous version of the path models. Results of these specified path models indicated a relation between gender and task performance but did not show relations between gender and the independent variables, nor the dependent variables other than task performance. Therefore, gender could be considered irrelevant for answering our specific research questions. Therefore, gender was not considered in subsequent steps of this study. Analyses were conducted in Mplus 8.10^71^ using a maximum likelihood estimator (MLR) for model estimation. Missing data, which accounted for 2.2% of data across all included variables, were handled using full-information maximum likelihood estimator (FIML). Little’s missing-completely-at-random (MCAR) test indicated no systematic patterns of missing values (*χ²* = 24.09, df = 45, *p* = 0.995).

## Data Availability

The datasets used and analyzed during the current study are available from the corresponding author on reasonable request.

## Code availability

The underlying code for this study is not publicly available but may be made available to qualified researchers on reasonable request from the corresponding author.
